# Age-Related Macular Degeneration and Incident Stroke: A Systematic Review and Meta-Analysis

**DOI:** 10.1371/journal.pone.0142968

**Published:** 2015-11-18

**Authors:** Antonio B. Fernandez, Gregory A. Panza, Benjamin Cramer, Saurav Chatterjee, Ramya Jayaraman, Wen-Chih Wu

**Affiliations:** 1 Division of Cardiology, Hartford Hospital, Hartford, Connecticut, United States of America; 2 University of Connecticut, School of Medicine, Farmington, CT, United States of America; 3 University of Connecticut, Department of Kinesiology, Storrs, CT, United States of America; 4 Frank H. Netter School of Medicine, Quinnipiac University, Hamden, CT, United States of America; 5 St Luke's-Roosevelt Hospitals of the Mount Sinai Health System, New York, NY, United States of America; 6 Department of Medicine, St. Vincent's Medical Center, Bridgeport, CT, United States of America; 7 Medical Service, Providence VA Medical Center and Department of Medicine, Alpert Medical School of Brown University, Providence, RI, United States of America; Tufts University, UNITED STATES

## Abstract

**Background:**

Age-related macular degeneration (AMD) is the leading cause of vision loss and blindness in people over 65 years old in the United States and has been associated with cardiovascular risk and decreased survival. There is conflicting data, however, regarding the contribution of AMD to the prediction of stroke.

**Aim:**

To determine whether AMD is a risk indicator for incident stroke in a meta-analysis of available prospective and retrospective cohort studies published in the English literature.

**Methods:**

We performed a systematic literature search of all studies published in English with Pub Med and other databases from 1966 to August 2014, reporting stroke incidence in patients with macular degeneration. Two investigators independently extracted the data. A random effects model was used to report Odds ratios (OR), with corresponding 95% confidence intervals (CI). Meta-regression using a mixed linear model was used to understand potential heterogeneity amongst studies.

**Results:**

We identified 9 studies that reported stroke incidence in patients with and without early AMD (N = 1,420,978). No significant association was found between early AMD with incident stroke. Combined, these 9 studies demonstrated random effects (OR, 1.12; CI, 0.86–1.47; I^2^ = 96%). Meta-regression on baseline covariates of age, sex, and year of publication did not significantly relate to heterogeneity.

**Conclusions:**

We found no significant relationship between AMD and incident stroke. Further studies are needed to clarify other causes of decreased survival in patients with AMD.

## Introduction

Age-related macular degeneration (AMD) is the degeneration of the center of the retina, or macula, which plays a major role in central vision. It constitutes the leading cause of vision loss and blindness in people ages 65 years and over in the United States, having affected more than 1.75 million individuals by the year 2000 [1]. As the American population grows older, the number of people affected by the disease is projected to increase to 3 million by the year 2020 [1]. AMD was initially suggested as a vascular disorder by Verhoeff and Grossman in 1937 [2], a hypothesis supported by several subsequent studies [3–6]. Although the precise etiology of AMD remains unclear, the disease has been associated with various traditional cardiovascular risk factors, such as age [7], elevated serum cholesterol levels [8], hypertension [9], and cigarette smoking [10], which raises the question as to whether AMD itself is an independent risk factor for cardiovascular disease (CVD) events. The specific relationship between AMD and stroke has not been clearly elucidated and the few available studies have yielded mixed results. Such divergent literature warranted a systematic review and meta-analysis. Thus, we conducted a systematic review of the literature and meta-analysis (S1 PRISMA Checklist) in order to evaluate the association between AMD and stroke.

## Methods

### Literature Search and Data Abstraction

We performed a systematic literature search of all studies published in English with PubMed, EMBASE, and the NIH Clinical Trials Registry from 1966 to July 2014 using the search terms: “Age-related macular degeneration”, “senile macular degeneration”, “maculopathy”, “retinal degeneration”, “retina”, and “disciform macular degeneration” in various combinations with the terms “cerebrovascular disease”, “apoplexy”, “cerebrovascular accident”, “brain vascular accident”, “ischemia”, and “stroke” (S1 File). Each term was then broken into its component parts and searched as individual terms as well. References of original and review articles were cross-checked. Study selection was performed by two independent reviewers (ABF, SC), divergences being resolved by consensus with the senior author (WCW). The second and third literature searches did not contribute additional studies for the analyses. Citations were first scanned at the title/abstract level. Shortlisted studies were then retrieved in full text.

### Selection Criteria and Data Extraction

We performed initial systematic screening of articles on the basis of the abstract and titles in November 2012, December 2012, and July 2014, with the reviewer in December 2012 being blinded to the initial search. We performed a full-text review of manuscripts selected. Relevant abstracts and articles were reviewed in detail. Selected citations from these articles were also reviewed.

Inclusion criteria for our meta-analysis were both retrospective and prospective observational studies performed in the last 50 years, comparing incident stroke in participants with and without AMD. Given the paucity of data, no studies were excluded. Studies comparing stroke event rates as a function of anti-angiogenic therapy for AMD were not included in the analyses given the lack of non-AMD cohorts. The primary outcome of interest was incident stroke, including ischemic and/or hemorrhagic strokes, or stroke-related death. Studies reporting early and late AMD and stroke incidence were reviewed. Most studies reporting late AMD were excluded due to extremely variable lengths of follow-ups, and in many instances, lack of specifications into stroke outcomes categorizing them into ischemic and hemorrhagic nature. However, among studies reporting both AMD categories, early AMD point estimates were included if they had similar length of follow-up. Adjusted risk ratios (RR) were included where available, however raw event rates or crude RR were used if adjusted RR were not available.

### Statistical Analysis

Review Manager 5.1 (Copenhagen: The Nordic Cochrane Centre, The Cochrane Collaboration, Denmark) and STATA version 11.2 SE were used for analysis. Outcomes were assessed using random effects models, to account for presence of possible significant heterogeneity (evaluated and quantified with the I^2^-statistic), and then pooled random effects odds ratios (OR) were calculated with 95% confidence intervals (CI) following the DerSimonian and Laird method. The quality of the studies was assessed on the basis of elements from the strengthening Meta-analysis of Observational Studies in Epidemiology (MOOSE) checklist for cohort studies [11]. We did not assign a threshold for study inclusion noting paucity of data.

## Results

**Fig 1:**
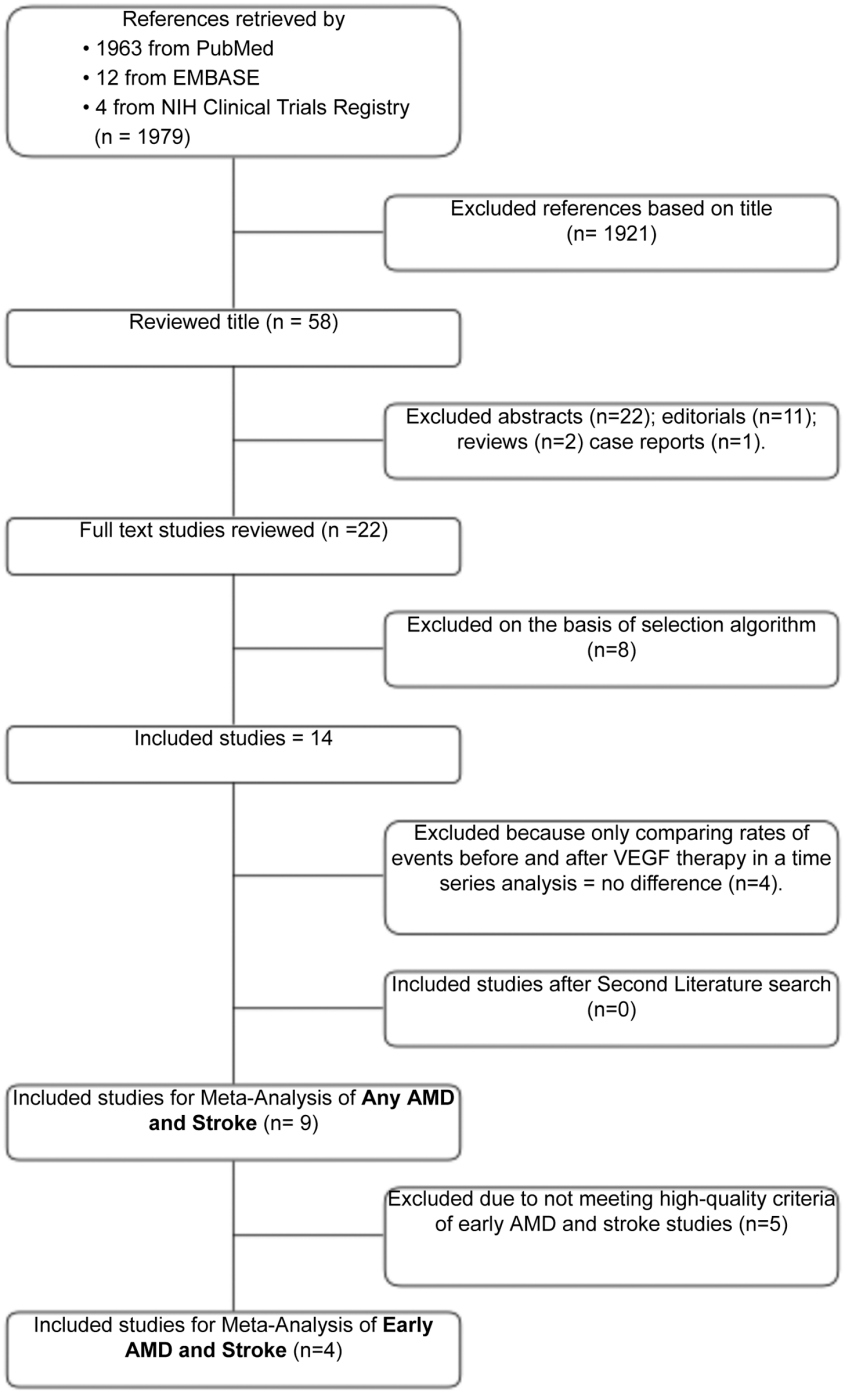
Flowchart of Article Selection for Meta-analysis of AMD and Stroke. Potentially relevant references identified and screened for retrieval. AMD, Age-related Macular degeneration; VEGF, Vascular endothelial growth factor.

Our search yielded 9 studies that directly evaluate the association between AMD and stroke events. The combined search of PubMed, EMBASE, and NIH clinical Trials registry retrieved 1979 citations. The flow chart in Fig 1 shows that 1921 articles were discarded after a title and abstract review. Thirty-six editorials, reviews and case reports were excluded for not meeting our observational criteria. Eight retrospective or prospective observational studies were excluded because they did not match the clinical question and 4 studies were excluded because of anti-VEGF treatment intervention. Overall, 9 studies enrolling a total of 1,420,978 patients were included in our analysis (S1 Data). Among these, 166,756 patients had any type of AMD and 1,254,222 had no AMD. 3,948 patients in the any AMD group and 2,259 in the no AMD group suffered strokes during their respected studies, and were thus considered primary end point in our analysis.

**Fig 2:**
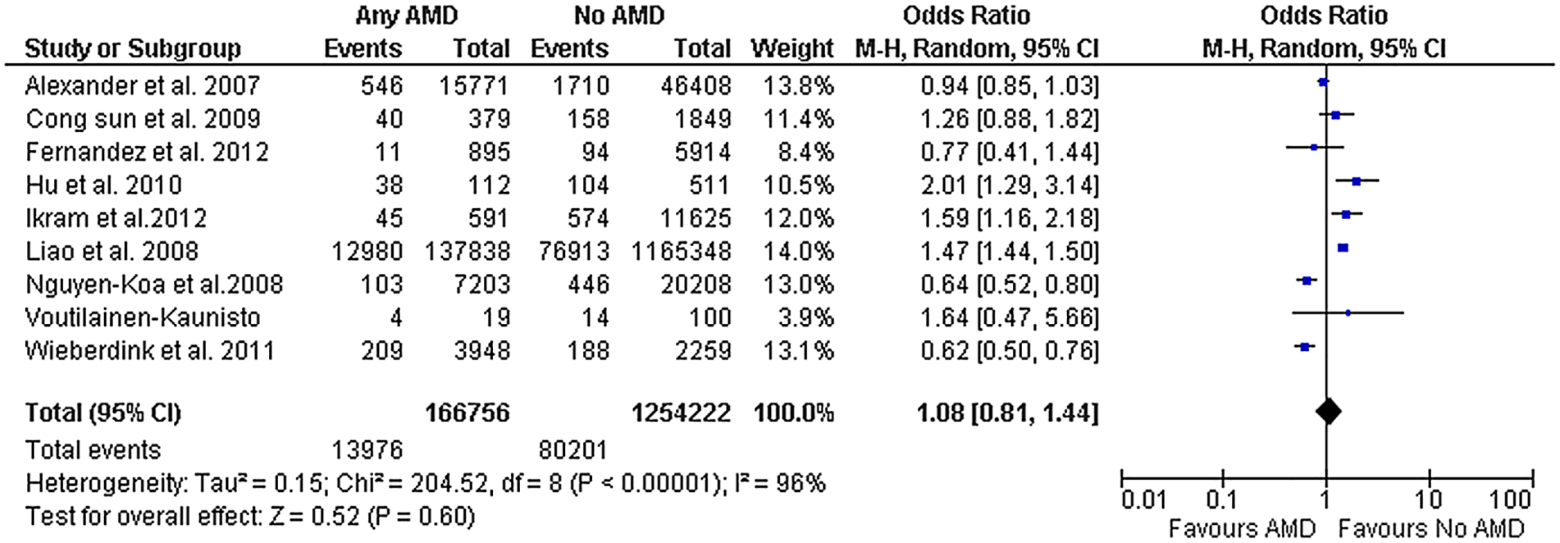
Any AMD and Stroke. Diamond indicates the overall summary for the analysis, width of the diamond represents the 95% CI (Confidence Interval), and boxes represent the weight of individual studies in the pooled analysis. Trials to the left of the vertical line showed a reduction in the risk of stroke events favored AMD; those to the right showed an increase in risk favored No AMD. Abbreviations: AMD, Age-related macular degeneration; M-H, Mantel-Haenszel.

The quality of these studies varied greatly. There were 4 retrospective studies and 5 prospective studies. All 4 retrospective studies ascertained both the exposure and the outcomes variables based on claim databases. The 5 prospective studies used retinal funduscopic examinations to determine AMD type and severity but only in 3 studies incident strokes were adjudicated by neurologists. One study used a different retinal photographs AMD grading system. Studies varied greatly in the time period used to ascertained for outcomes (ranging from 2 to 13.6 years). Most of the studies had similar gender composition. Only 5 studies evaluated the relationship between early AMD and stroke (Table 1). Of those, only 1 study was excluded in our sub-group analysis because stroke status was self-reported. Fig 2 shows the results of the 9 enrolled studies, demonstrating no statistically significant association between AMD with incident stroke, random effects (OR, 1.12; CI, 0.86–1.47; p = 0.40). Significant heterogeneity was noted (I^2^ = 96%). However, meta-regression on baseline covariates of age, sex, and duration of follow up failed to identify the source of the heterogeneity.

**Table 1:** Characteristics of included studies that evaluated the associations of AMD and risk of stroke.

Study	Year	N	Race	Mean age (yr)	Female (%)	Time period (yr)	AMD	Multivariate analyses	Stroke Assessment	Type of study	AMD assessment
Voutilainen-Kaunisto et al [30]	2000	277	C	55	66	10	Not specified	Not reported	Review of medical records	Prospective	Fundus Photography
Alexander et al [15]	2007	62,179	C	75	59	2	Only neovascular	Comorbidities by select Charlson-Deyo categories, HLD and HTN	Claim data	Retrospective	Claim data
Nguyen-Koa et al [18]	2008	27,411	-	75	59.4	3.4	Only neovascular	Angina, cardiac arrhythmia, Charlson score, congestive heart failure, diabetes, heart disease, history of acute MI, history of CVA, hyperlipidemia, hypertension, and other cerebrovascular disease.	Claim data	Retrospective	Claim data
Liao et al [31]	2008	1,303,186	C	75	60	2	Neovascular and non-neovascular	Age group, gender, race, hypertension, and diabetes	Claim data	Retrospective	Claim data
Sun et al [17]	2009	2228	C, AA	79.9	64.7	6	Early and Late	Age, sex, ethnicity, SBP and DBP, HTN, fasting glucose, diabetes, TGA, smoking, LDL, CRP	Adjudicated after physician review of medical records	Prospective	Fundus Photography
Hu et al [32]	2010	1,254	A	62.7	39.7	5	Neovascular	Age, sex, HTN, DM, CHD, HLD, renal disease, income, urbanization lavel, and geographic region.	Claim data	Retrospective	Claim data
Wieberdink et al [33]	2011	6,207	C	67.6	59.6	13.6	Neovascular and non-neovascular	Age and sex, diabetes, SBP, antihypertensive medication, smoking, cholesterol, HDL, carotid artery plaques, BMI, alcohol intake and CRP	Self-reported verified with medical records by neurologists	Prospective	Fundus Photography
Ikram et al [34]	2012	12,216	C, AA	59.9	55.8	13	Early and Late	Age, sex, race, field, center, mean BP, antihypertensive medications, fasting glucose, cholesterol, HDL, TGA, BMI, atrial fibrillation, white blood cell count, smoking, and alcohol consumption	Self-reported verify against discharge list	Prospective	Fundus Photography
Fernandez et al [35]	2012	6,233	C, AA, A	62	52.4	5.4	Early and Late	Race, HTN, smoking, site, CRP, education, DM, BMI, cholesterol, LDL	Adjudicated by after neurologist review of medical record	Prospective	Fundus Photography

Abbreviations: C = Caucasian; AA = African American; A = Asian; MI = Myocardial infarction; CVA = Cerebrovascular accident; SBP = Systolic blood pressure; DBP = Diastolic blood pressure; HTN = Hypertension; TGA = Transient global amnesia; LDL = Low-density lipoprotein; CRP = C-reactive protein; DM = Diabetes mellitus; CHD = Coronary heart disease; HLD = Hyperlipidemia; HDL = High-density lipoprotein; BMI = Body mass index.

### Meta-Analysis: Subgroup Analysis

**Fig 3:**
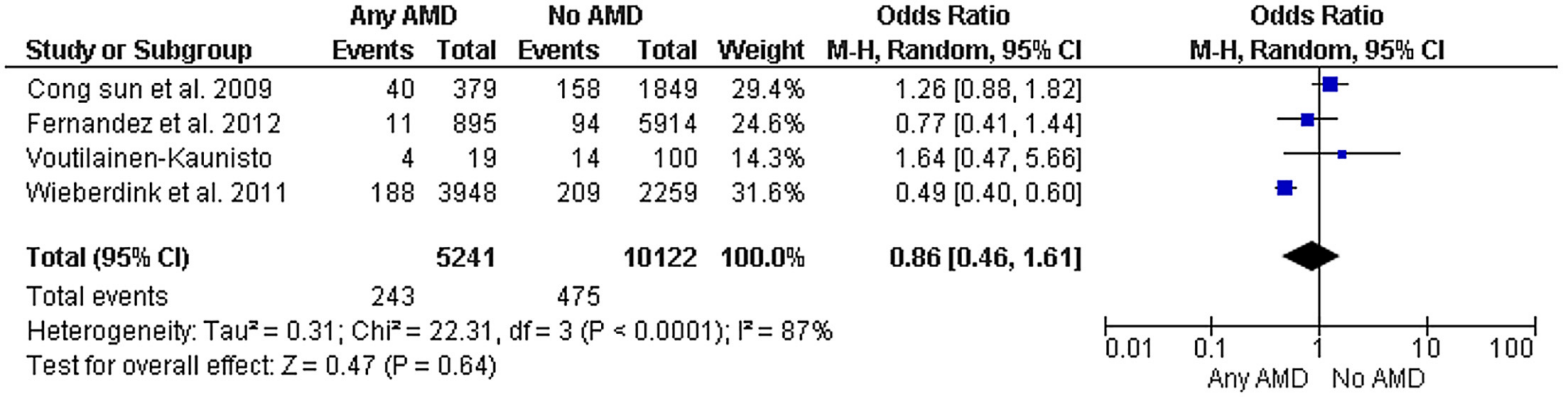
High-quality Studies of AMD and risk of stroke. Diamond indicates the overall summary for the analysis, width of the diamond represents the 95% CI (Confidence Interval), and boxes represent the weight of individual studies in the pooled analysis. Trials to the left of the vertical line showed a reduction in the risk of stroke events favored AMD; those to the right showed an increase in risk favored No AMD. Abbreviations: AMD, Age-related macular degeneration; M-H, Mantel-Haenszel.

When grouped by early vs. late AMD, 6 studies addressed the role of early AMD and stroke incidence (N = 93,756) and showed similar results, with no statistically significant relationship between early AMD and stroke (Fig 3). These studies included a total of 2,197 patients with early AMD and 72,559 had no AMD; 889 patients suffered stroke in the early AMD group compared to 2,952 patients in the no AMD group. The pooled random effects OR was 1.30 (CI, 0.90–1.89, p = 0.16) with I^2^ = 92%.

Fig 3 shows results of studies considered high in quality defined as prospective, using retinal photographs and confirmed strokes by medical records. High-quality studies are congruent with the overall results demonstrating no increase in odds of stroke with AMD (OR, 0.86; CI, 0.46–1.61; I^2^ = 87%).

## Discussion

Our systematic review and meta-analysis of 9 studies, did not find a significant relationship between AMD and incident stroke. Even though there seems to be an increased CVD mortality and event rates in patients with more severe types of AMD in some studies,[12,13,14] this has not been supported by findings across all studies [15,16,17,18]. Very few studies relate AMD to the development of strokes, and their results have been conflicting [13, 15]. Subgroup analysis of high-quality studies looking at early AMD and incident stroke also failed to find a significant relationship. An odds ratio of 1.08 was found between AMD and incident stroke, but wide divergence in study results contributed to our confidence interval crossing unity. The non-significant association between AMD and stroke may arise from several sources. Overall, the assessment of the relationship between AMD and stroke might require a longer follow-up period. Inconsistencies between studies might also have arisen from the undocumented use of antiangiogenic therapy for the treatment of neovascular AMD. Since its approval for the treatment of neovascular AMD late in 2004, the safety of intravitreal injections of anti-VEGF agents has been controversial given the potential for increased risk of atherothrombotic events when these agents are used systemically. To this date, no clear consensus about whether this treatment strategy used locally in the eye have clinical deleterious effects on the cardiovascular system[19]. Nonetheless, most of the current data tend to indicate no significant differences in mortality, stroke or MI risk between anti-VEGF therapy and the other treatment modalities [20].Potential biases and shortcomings were present in the included studies. First, adjustments for important confounders such as reticular macular disease (RMD) were not included. Current advances in clinical imaging, including autofluorescence, near infrared reflectance, indocyanine green angiography and high-resolution spectral domain optical coherence tomography (SD-OCT) suggest that RMD is another major pathway to advanced AMD. RMD, with characteristic reticular patterns on en face imaging, appears to involve the retinal pigment epithelium and choriocapillaris [21], and also presents with subretinal drusenoid deposits on histopathology and SD-OCT [22, 23]. RMD is far more prevalent and confers a worse prognosis than previously realized [24, 25]. It has been prospectively associated with double the rate of onset of both choroidal neovascularization (CNV) [24, 26] and geographic atrophy (GA) than soft drusen and is highly prevalent in and spatially predictive value for the development of GA [25], particularly in the most common multilobular form. If the choroid is indeed affected in RMD, a possible linkage with other systemic vascular diseases would be consistent [27, 28]. The high-risk phenotype RMD was not considered in any of the studies in this meta-analysis, suggesting that associations of AMD with stroke should be tested again with RMD in mind. Smoking status was another confounder not included in some of the analysis [15]. Furthermore, multivariate adjustment analysis models were used in most, but not all of the studies (Table 1). The more prevalent non-neovascular AMD was not included in some studies [15]. It is conceivable that qualitative biases remained unmeasured, affecting our estimates. This is particularly relevant for the studies using claim based data, which are affected by limitations inherent to the retrospective design and the limited accuracy of healthcare claims to define chronic conditions. In addition, some of the studies found in our search, had a relatively short follow-up period, which affects the number of events recorded and point estimates.

AMD has been attributed to excessive exposure to light (18), dietary deficiencies, and oxidative stress [29]. These theories, however, do not fully explain all the pathologic findings of AMD. A more plausible vascular model proposes that AMD is the result of hemodynamic changes in the choroidal vasculature and sclerosis of the sclera and Bruch membrane [24]. Lipid deposition, and age related collagen and elastic tissue degeneration result in sclerosis and stiffening of the sclera and Bruch membrane with luminal narrowing of the choriocapillaris [24]. Angiographic studies and spectral Doppler examinations of the short posterior ciliary arteries also support this hypothesis. Were AMD a strong predictor of stroke events, its presence could have significant implications for disease prevention and might help ongoing efforts at refining individual stroke risk prediction estimates. Further studies that surpass the limitations described in this meta-analysis are called for. Both diseases constitute major public health issues in the United States and worldwide. If this hypothesis were to be confirmed, a subgroup of patients with early signs of AMD might be targeted for a more aggressive risk factor modification and prevention.

Given the similarities between age-related macular degeneration and stroke at the pathophysiologic and genetic levels, AMD is attracting increasing interest within the cardiovascular community. AMD may have the potential to identify a subgroup of patients at an increased risk of CAD and stroke, improving primary prevention efforts. However, the clinical evidence linking AMD and stroke remains inconclusive, with additional research being necessary to fully characterize this potentially important relationship.

## Supporting Information

S1 Data Data for studies included in Meta-analysis. (XLS)

S1 File Search terms used for systematic literature search. (DOCX)

S1 PRISMAChecklist 2009 PRISMA Checklist. (DOC)
