# Supplementary material for: Age-Related Macular Degeneration and Incident Stroke: A Systematic Review and Meta-Analysis
Source: PLoS One. 2015 Nov 18;10(11):e0142968. doi: 10.1371/journal.pone.0142968 (PMC4651536; doi:10.1371/journal.pone.0142968)
Supplement: S1 File — (DOCX) [file pone.0142968.s002.docx]

(Cerebrovascular Disorders OR cerebrovascular disorder OR cardiovascular diseases OR cardiovascular disease OR (heart disease OR heart diseases AND (ischemia OR ischemic OR ischaemia)) OR myocardial ischemia OR coronary disease OR coronary diseases OR coronary artery disease OR ischemic heart disease OR stroke OR apoplexy OR brain vascular accident OR brain vascular accidents OR cerebrovascular accident OR cerebrovascular accidents) AND (((Retinal degeneration OR retina) AND (age related OR macular OR senile macular OR disciform macular)) OR macular degeneration OR age related macular degeneration OR senile macular degeneration OR disciform macular degeneration) NOT (Diabetes Mellitus OR diabetes OR diabetic)

(("cerebrovascular disorders"[MeSH Terms] OR ("cerebrovascular"[All Fields] AND "disorders"[All Fields]) OR "cerebrovascular disorders"[All Fields]) OR ("cerebrovascular disorders"[MeSH Terms] OR ("cerebrovascular"[All Fields] AND "disorders"[All Fields]) OR "cerebrovascular disorders"[All Fields] OR ("cerebrovascular"[All Fields] AND "disorder"[All Fields]) OR "cerebrovascular disorder"[All Fields]) OR ("cardiovascular diseases"[MeSH Terms] OR ("cardiovascular"[All Fields] AND "diseases"[All Fields]) OR "cardiovascular diseases"[All Fields]) OR ("cardiovascular diseases"[MeSH Terms] OR ("cardiovascular"[All Fields] AND "diseases"[All Fields]) OR "cardiovascular diseases"[All Fields] OR ("cardiovascular"[All Fields] AND "disease"[All Fields]) OR "cardiovascular disease"[All Fields]) OR (("heart diseases"[MeSH Terms] OR ("heart"[All Fields] AND "diseases"[All Fields]) OR "heart diseases"[All Fields] OR ("heart"[All Fields] AND "disease"[All Fields]) OR "heart disease"[All Fields]) OR ("heart diseases"[MeSH Terms] OR ("heart"[All Fields] AND "diseases"[All Fields]) OR "heart diseases"[All Fields]) AND (("ischaemia"[All Fields] OR "ischemia"[MeSH Terms] OR "ischemia"[All Fields]) OR ("ischemia"[MeSH Terms] OR "ischemia"[All Fields] OR "ischemic"[All Fields]) OR ("ischaemia"[All Fields] OR "ischemia"[MeSH Terms] OR "ischemia"[All Fields]))) OR ("myocardial ischaemia"[All Fields] OR "myocardial ischemia"[MeSH Terms] OR ("myocardial"[All Fields] AND "ischemia"[All Fields]) OR "myocardial ischemia"[All Fields] OR "coronary artery disease"[MeSH Terms] OR ("coronary"[All Fields] AND "artery"[All Fields] AND "disease"[All Fields]) OR "coronary artery disease"[All Fields] OR ("myocardial"[All Fields] AND "ischemia"[All Fields])) OR ("coronary disease"[MeSH Terms] OR ("coronary"[All Fields] AND "disease"[All Fields]) OR "coronary disease"[All Fields]) OR ("coronary disease"[MeSH Terms] OR ("coronary"[All Fields] AND "disease"[All Fields]) OR "coronary disease"[All Fields] OR ("coronary"[All Fields] AND "diseases"[All Fields]) OR "coronary diseases"[All Fields]) OR ("coronary artery disease"[MeSH Terms] OR ("coronary"[All Fields] AND "artery"[All Fields] AND "disease"[All Fields]) OR "coronary artery disease"[All Fields]) OR ("ischaemic heart disease"[All Fields] OR "myocardial ischemia"[MeSH Terms] OR ("myocardial"[All Fields] AND "ischemia"[All Fields]) OR "myocardial ischemia"[All Fields] OR ("ischemic"[All Fields] AND "heart"[All Fields] AND "disease"[All Fields]) OR "ischemic heart disease"[All Fields] OR "coronary artery disease"[MeSH Terms] OR ("coronary"[All Fields] AND "artery"[All Fields] AND "disease"[All Fields]) OR "coronary artery disease"[All Fields] OR ("ischemic"[All Fields] AND "heart"[All Fields] AND "disease"[All Fields])) OR ("stroke"[MeSH Terms] OR "stroke"[All Fields]) OR ("stroke"[MeSH Terms] OR "stroke"[All Fields] OR "apoplexy"[All Fields]) OR ("stroke"[MeSH Terms] OR "stroke"[All Fields] OR ("brain"[All Fields] AND "vascular"[All Fields] AND "accident"[All Fields]) OR "brain vascular accident"[All Fields]) OR ("stroke"[MeSH Terms] OR "stroke"[All Fields] OR ("brain"[All Fields] AND "vascular"[All Fields] AND "accidents"[All Fields]) OR "brain vascular accidents"[All Fields]) OR ("stroke"[MeSH Terms] OR "stroke"[All Fields] OR ("cerebrovascular"[All Fields] AND "accident"[All Fields]) OR "cerebrovascular accident"[All Fields]) OR ("stroke"[MeSH Terms] OR "stroke"[All Fields] OR ("cerebrovascular"[All Fields] AND "accidents"[All Fields]) OR "cerebrovascular accidents"[All Fields])) AND (((("retinal degeneration"[MeSH Terms] OR ("retinal"[All Fields] AND "degeneration"[All Fields]) OR "retinal degeneration"[All Fields]) OR ("retina"[MeSH Terms] OR "retina"[All Fields])) AND ((("Age"[Journal] OR "age"[All Fields] OR "Age (Omaha)"[Journal] OR "age"[All Fields] OR "Age (Dordr)"[Journal] OR "age"[All Fields] OR "Adv Genet Eng"[Journal] OR "age"[All Fields]) AND related[All Fields]) OR macular[All Fields] OR (senile[All Fields] AND macular[All Fields]) OR (disciform[All Fields] AND macular[All Fields]))) OR ("macular degeneration"[MeSH Terms] OR ("macular"[All Fields] AND "degeneration"[All Fields]) OR "macular degeneration"[All Fields]) OR ("macular degeneration"[MeSH Terms] OR ("macular"[All Fields] AND "degeneration"[All Fields]) OR "macular degeneration"[All Fields] OR ("age"[All Fields] AND "related"[All Fields] AND "macular"[All Fields] AND "degeneration"[All Fields]) OR "age related macular degeneration"[All Fields]) OR ("macular degeneration"[MeSH Terms] OR ("macular"[All Fields] AND "degeneration"[All Fields]) OR "macular degeneration"[All Fields] OR ("senile"[All Fields] AND "macular"[All Fields] AND "degeneration"[All Fields]) OR "senile macular degeneration"[All Fields]) OR (disciform[All Fields] AND ("macular degeneration"[MeSH Terms] OR ("macular"[All Fields] AND "degeneration"[All Fields]) OR "macular degeneration"[All Fields]))) NOT (("diabetes mellitus"[MeSH Terms] OR ("diabetes"[All Fields] AND "mellitus"[All Fields]) OR "diabetes mellitus"[All Fields]) OR ("diabetes mellitus"[MeSH Terms] OR ("diabetes"[All Fields] AND "mellitus"[All Fields]) OR "diabetes mellitus"[All Fields] OR "diabetes"[All Fields] OR "diabetes insipidus"[MeSH Terms] OR ("diabetes"[All Fields] AND "insipidus"[All Fields]) OR "diabetes insipidus"[All Fields]) OR diabetic[All Fields]) AND (("2013/01/01"[PDAT] : "2014/12/31"[PDAT]) AND "humans"[MeSH Terms] AND English[lang])

| Cerebrovascular Disorders | "cerebrovascular disorders"[MeSH Terms] OR ("cerebrovascular"[All Fields] AND "disorders"[All Fields]) OR "cerebrovascular disorders"[All Fields] |
| --- | --- |
| cerebrovascular disorder | "cerebrovascular disorders"[MeSH Terms] OR ("cerebrovascular"[All Fields] AND "disorders"[All Fields]) OR "cerebrovascular disorders"[All Fields] OR ("cerebrovascular"[All Fields] AND "disorder"[All Fields]) OR "cerebrovascular disorder"[All Fields] |
| cardiovascular diseases | "cardiovascular diseases"[MeSH Terms] OR ("cardiovascular"[All Fields] AND "diseases"[All Fields]) OR "cardiovascular diseases"[All Fields] |
| cardiovascular disease | "cardiovascular diseases"[MeSH Terms] OR ("cardiovascular"[All Fields] AND "diseases"[All Fields]) OR "cardiovascular diseases"[All Fields] OR ("cardiovascular"[All Fields] AND "disease"[All Fields]) OR "cardiovascular disease"[All Fields] |
| heart disease | "heart diseases"[MeSH Terms] OR ("heart"[All Fields] AND "diseases"[All Fields]) OR "heart diseases"[All Fields] OR ("heart"[All Fields] AND "disease"[All Fields]) OR "heart disease"[All Fields] |
| heart diseases | "heart diseases"[MeSH Terms] OR ("heart"[All Fields] AND "diseases"[All Fields]) OR "heart diseases"[All Fields] |
| ischemia | "ischaemia"[All Fields] OR "ischemia"[MeSH Terms] OR "ischemia"[All Fields] |
| ischemic | "ischemia"[MeSH Terms] OR "ischemia"[All Fields] OR "ischemic"[All Fields] |
| ischaemia | "ischaemia"[All Fields] OR "ischemia"[MeSH Terms] OR "ischemia"[All Fields] |
| myocardial ischemia | "myocardial ischaemia"[All Fields] OR "myocardial ischemia"[MeSH Terms] OR ("myocardial"[All Fields] AND "ischemia"[All Fields]) OR "myocardial ischemia"[All Fields] OR "coronary artery disease"[MeSH Terms] OR ("coronary"[All Fields] AND "artery"[All Fields] AND "disease"[All Fields]) OR "coronary artery disease"[All Fields] OR ("myocardial"[All Fields] AND "ischemia"[All Fields]) |
| coronary disease | "coronary disease"[MeSH Terms] OR ("coronary"[All Fields] AND "disease"[All Fields]) OR "coronary disease"[All Fields] |
| coronary diseases | "coronary disease"[MeSH Terms] OR ("coronary"[All Fields] AND "disease"[All Fields]) OR "coronary disease"[All Fields] OR ("coronary"[All Fields] AND "diseases"[All Fields]) OR "coronary diseases"[All Fields] |
| coronary artery disease | "coronary artery disease"[MeSH Terms] OR ("coronary"[All Fields] AND "artery"[All Fields] AND "disease"[All Fields]) OR "coronary artery disease"[All Fields] |
| ischemic heart disease | "ischaemic heart disease"[All Fields] OR "myocardial ischemia"[MeSH Terms] OR ("myocardial"[All Fields] AND "ischemia"[All Fields]) OR "myocardial ischemia"[All Fields] OR ("ischemic"[All Fields] AND "heart"[All Fields] AND "disease"[All Fields]) OR "ischemic heart disease"[All Fields] OR "coronary artery disease"[MeSH Terms] OR ("coronary"[All Fields] AND "artery"[All Fields] AND "disease"[All Fields]) OR "coronary artery disease"[All Fields] OR ("ischemic"[All Fields] AND "heart"[All Fields] AND "disease"[All Fields]) |
| stroke | "stroke"[MeSH Terms] OR "stroke"[All Fields] |
| apoplexy | "stroke"[MeSH Terms] OR "stroke"[All Fields] OR "apoplexy"[All Fields] |
| brain vascular accident | "stroke"[MeSH Terms] OR "stroke"[All Fields] OR ("brain"[All Fields] AND "vascular"[All Fields] AND "accident"[All Fields]) OR "brain vascular accident"[All Fields] |
| brain vascular accidents | "stroke"[MeSH Terms] OR "stroke"[All Fields] OR ("brain"[All Fields] AND "vascular"[All Fields] AND "accidents"[All Fields]) OR "brain vascular accidents"[All Fields] |
| cerebrovascular accident | "stroke"[MeSH Terms] OR "stroke"[All Fields] OR ("cerebrovascular"[All Fields] AND "accident"[All Fields]) OR "cerebrovascular accident"[All Fields] |
| cerebrovascular accidents | "stroke"[MeSH Terms] OR "stroke"[All Fields] OR ("cerebrovascular"[All Fields] AND "accidents"[All Fields]) OR "cerebrovascular accidents"[All Fields] |
| Retinal degeneration | "retinal degeneration"[MeSH Terms] OR ("retinal"[All Fields] AND "degeneration"[All Fields]) OR "retinal degeneration"[All Fields] |
| retina | "retina"[MeSH Terms] OR "retina"[All Fields] |
| age | "Age"[Journal] OR "age"[All Fields] OR "Age (Omaha)"[Journal] OR "age"[All Fields] OR "Age (Dordr)"[Journal] OR "age"[All Fields] OR "Adv Genet Eng"[Journal] OR "age"[All Fields] |
| macular degeneration | "macular degeneration"[MeSH Terms] OR ("macular"[All Fields] AND "degeneration"[All Fields]) OR "macular degeneration"[All Fields] |
| age related macular degeneration | "macular degeneration"[MeSH Terms] OR ("macular"[All Fields] AND "degeneration"[All Fields]) OR "macular degeneration"[All Fields] OR ("age"[All Fields] AND "related"[All Fields] AND "macular"[All Fields] AND "degeneration"[All Fields]) OR "age related macular degeneration"[All Fields] |
| senile macular degeneration | "macular degeneration"[MeSH Terms] OR ("macular"[All Fields] AND "degeneration"[All Fields]) OR "macular degeneration"[All Fields] OR ("senile"[All Fields] AND "macular"[All Fields] AND "degeneration"[All Fields]) OR "senile macular degeneration"[All Fields] |
| Diabetes Mellitus | "diabetes mellitus"[MeSH Terms] OR ("diabetes"[All Fields] AND "mellitus"[All Fields]) OR "diabetes mellitus"[All Fields] |
| diabetes | "diabetes mellitus"[MeSH Terms] OR ("diabetes"[All Fields] AND "mellitus"[All Fields]) OR "diabetes mellitus"[All Fields] OR "diabetes"[All Fields] OR "diabetes insipidus"[MeSH Terms] OR ("diabetes"[All Fields] AND "insipidus"[All Fields]) OR "diabetes insipidus"[All Fields] |
| Humans[Mesh] | "humans"[MeSH Terms] |
